# The Impact of Private Standards on Corporate Social Responsibility Compliance and Rural Workers' Motivation in Developing Countries: A Study of Mango Farms in Pakistan

**DOI:** 10.1155/2021/9985784

**Published:** 2021-08-24

**Authors:** Honglei Tang, Zeeshan Rasool, Ahmad Imran Khan, Anum Afzal Khan, Mohsin Ali Khan, Ghulam Ahmad Azaz

**Affiliations:** ^1^School of Economics and Management, Huzhou University, China; ^2^School of Economics and Management, Shaanxi University of Science and Technology, Xi'an 710021, China; ^3^Putra Business School, University of Putra, (UPM), 43400 Serdang, Selangor, Malaysia; ^4^National University of Modern Languages Multan Campus, Pakistan; ^5^Department of Business, Management & Law, Cardiff Metropolitan University, Llandaff Campus, Western Ave, Cardiff CF5 2YB, UK

## Abstract

This study examines the role of a private standard on corporate social responsibility (CSR) compliance in the Pakistani mango industry and how this compliance affects rural workers' motivation. Pakistan is the fifth largest mango producer in the world and the fourth largest exporter in global mango trade; also, mango is the biggest fruit crop within the country. Mango trade is subject to trade terms, where buyers decide the conditions of trade agreements by means of codes of conduct. The key dimensions of the codes involved in agrofood trade are food safety, traceability, worker welfare, and environmental consideration, issues which are all connected with CSR. Private standards ensure compliance with these codes of conduct. This study draws on interviews and a questionnaire survey with certified mango producers and farm workers in Pakistan. The mango industry also involves other stakeholders such as government institutes and NGOs; interviews were also conducted with their representatives. Given that this study is an impact assessment research, the researcher designed a theoretical framework using a mixed method approach to investigate the rationale behind acquiring the standard by the mango growers in Pakistan and what impact (if any) this shift has generated with regard to the farm workers' job satisfaction and motivation. This study is the first to empirically examine good agricultural practices in Pakistan and evaluate their impact. This study shows that private standards play a significant role in ensuring compliance, and CSR practices implemented through them were found to be positively related to the rural workers' job satisfaction and motivation. Furthermore, this study has made separate contributions to theory, methodology, and practice. The production of the synergistic model for improving compliance is among the key highlights of the study. The findings of this study can extend to other agriculture and primary production industry workers in Pakistan and even beyond to other developing countries' rural agriculture workers.

## 1. Introduction

Since the 1990s, labour conditions have been addressed and looked after with the help of corporate voluntary codes of conduct. There has been a shift from transnational corporation (TNC) regulations set by governments towards corporate self-regulation. Businesses in developed countries and their associates (producers, growers, or manufacturers) in developing countries are now expected to address social and environmental issues rather than relying on governments alone (Jenkins, Pearson and Seyfang, 2002). Businesses in developing countries are becoming global producers. Liberalisation of the international market enables small businesses to participate in global trade. However, all the interested businesses are required to comply with standards set by buyers in developed countries. Civil society and consumers in developed countries are becoming increasingly concerned about the production process of the goods they buy, as there have been several news stories published about the exploitation of workers, child labour, environment degradation, and other social issues in developing countries [1].

In 1997 EUREP, a working group based on 13 European megaretailers set up a technical standards committee and a steering committee in order to negotiate a joint resolution by integrating the various systems for supplying safe food. They formed a standard for good agricultural practices (GAP) and named it Eurep GAP (now GlobalGAP) which is required to be adopted by all global suppliers and was subject to audits [2]. Good agricultural practices (GAP) are voluntary private standards and codes. They have been developed during the last two decades by the food industry businesses (supermarkets and retail groups), producers, governments, and NGOs. The purpose was to systemise agriculture practices at the farm level to ensure food safety, quality, environment protection, better use of resources, and worker health and safety and welfare. Among the benefits of complying with GAP standards are better product quality and the facilitation of market access (FAO, 2007).

As the organisations who have designed GAP and other similar codes were based in developed countries, they faced huge criticism. Some of the major criticism states that these codes are only beneficial for buyers in developed countries, and they want to implement them to satisfy their consumer base [3], improvements in working and environmental conditions of workers are limited, these codes mainly encourage the large-scale producers as small-scale producers cannot afford the associated cost, and producers and other stakeholders in developing countries have never been consulted while formulating these codes (Lund-Thomsen, 2008; 2009; Nelson et al., 2007).

Codes of conduct in global trade are quasimandatory, and almost all trade contracts contain codes of business conduct which are formulated by the importing firms. These codes have a huge impact on exporting businesses in developing economies (Lee, Gereffi and Beauvais, 2012). As these codes and standards are becoming prerequisites [4], every farm or producer who wishes to enter high-value global chains is bound to face those challenges associated with such practices. Despite the rise in demands to do more [5], fewer efforts or interest has been shown by governments to provide exporters with the required knowledge and skills on how to achieve the required standards [6]. The codes of conduct in agriculture trade implemented through private standards are based on a corporate social responsibility (CSR) agenda for sustainable agriculture. Producers in developing countries need support in order to handle the pressure associated with codes of conduct and compliance with standards [7].

To meet the standards, there is an upgrade required in the infrastructure, management structure, and skills. It is generally considered that if developing countries could cope with the pressure by taking the right steps and equipping local producers and suppliers with the necessary training, it could lead to greater trading opportunities in the global value chain (Sun and Zhang, 2009). It is argued that governments in developing countries and international bodies must enter into joint ventures with producers and suppliers with the training and support as CSR is becoming a key component. The producers require support for capacity building to match the requirements from international standards [6].

Although all the aforementioned are the core issues of CSR, somehow, they apply fully or partially dependant on the nature of the business. Also, it depends on buyers' demands too, for example, the emergence of the UK antibribery act recently. Subsequently, all producers and suppliers seeking business terms with UK-based buyers are asked to sign a contract with the added clauses of antibribery practices (Ministry of Justice, 2010).

### 1.1. Problem Statement and Existing Gap

The research problem focuses on the impacts of private standard certification on the Pakistani mango industry and the rural farm workers, who worked at the certified farms. This study has successfully established quantifiable benefits of private standards by correlating their codes of conduct with the agricultural workers' motivation in the context of developing countries, as there were unanswered questions about the usefulness of private standards and what difference they make for poor farmers and workers [7].

There was no literature available about private standards and CSR in Pakistani agriculture apart from some articles about the Sialkot sports manufacturing industry and leather industry (Lund-Thomsen, 2008; 2009; 2013; Lund-Thomsen and Coe, 2015; [5, 8]) as well as a few UN and FAO publications. The Pakistani mango industry is going through a major shift, and GlobalGAP (a voluntary private standard) is the key player in this change. Currently, GlobalGAP is the only major private standard that is working in Pakistan. Thus far, no research has been done to investigate the rationale behind acquiring a GlobalGAP certification among Pakistani mango growers and furthermore how this affects the income and working conditions of workers and consequently the role this plays in worker job satisfaction and motivation.

Robinson [9] reported poor working conditions for workers in developing countries, even when CSR policies were implemented. There remained issues of forced labour and working in hazardous conditions. There has been continuous pressure from buyers to reduce costs, and this pressure forces producers to create a downward spiral of overall poor working conditions. Despite a huge shift in the sourcing practices and involvement of private standards in recent decades, little is known about the actual impact of such standards on the environment, income, and working conditions at the producers' end [7]. This particular gap forms the basis for one of the research questions of this research: what difference does the GlobalGAP certification make for the income, work, and environmental conditions of mango farmers and on-farm workers in Pakistan? Lemeilleur (2013) reports the adoption of the GlobalGAP program among smallholder mango producers in Peru; her study suggested a need for further investigation to assess the impact of GlobalGAP adoption on income and rural poverty.

The International Trade Centre has conducted a series of literature review analyses on the issue of private standards and their impact; the detailed and systematic review of existing literature identified the gaps, where literature is either missing or hazy. The report suggested that the impact assessment in the form of quantitative statistical data is missing, as it argued that most of the literature is based on conceptual and theoretical papers and fewer empirical studies have been carried out on this particular issue. The report further claimed that the majority of empirical studies had been done either in South America or in Africa [7].

There are several misconceptions about the role of private standards. The majority of studies focus on their impact on the financial gain and profitability of the participating growers or firms (Kersting and Wollni, 2012; [10]). However, when these private standards were initiated, especially the GlobalGAP, the main purpose was to ensure food safety, traceability, and sustainability (Forsman-Hugg et al., 2013; Hachez and Wouters, 2011). Although all private standards which work in food production and food processing have different codes of conduct, almost every standard has some commonalities. They all promised to protect the environment, to eliminate child labour, and to protect the core human rights of workers, such as their health, safety, and welfare (Forsman-Hugg et al., 2013).

There is an ongoing debate about the impact of private standards on developing countries' producers and workers; existing literature on the issue is underdeveloped and divided [7]. There was a growing body of evidence that a gap exists in the literature between buyers' CSR codes of practice and what is being experienced by stakeholders involved in mango production in Pakistan. This research is aimed at targeting this gap by examining CSR practices at the GlobalGAP certified mango farms to analyse its impact and to what extent it has improved the income and working conditions of the farm workers. The study further investigates the relationship between CSR practices and rural workers' job satisfaction and motivation.

### 1.2. Research Aim

The aim of this research was to examine CSR compliance and its effects on rural workers' motivation and job satisfaction at GlobalGAP certified mango farms in Pakistan.

### 1.3. Research Objectives

The following were the research objectives of this study:

- To synthesise the relevant literature on CSR practices in GVCs (global value chains), private standard certification, workers' conditions, social and environmental issues, and job satisfaction and motivation
- To develop a conceptual framework that characterises the relationship between CSR compliance, farm worker socioenvironmental conditions, and farm workers' job satisfaction and motivation
- To investigate the impact of GlobalGAP adoption on the Pakistani mango industry
- To examine CSR practices at the GlobalGAP certified mango farms in Punjab, Pakistan
- To quantify the CSR compliance and farm worker socioenvironmental conditions and identify CSR practices that have the most influence on the job satisfaction and motivation of agricultural workers
- To produce a new model and propose recommendations for changes in existing standards which will help to improve compliance

## 2. Literature Review

### 2.1. Corporate Social Responsibility

Corporate social responsibility (CSR) is a popular subject among academics and practitioners, albeit the core dimensions are economic, social, and environmental, commonly known as profit, people, and planet, also known as the triple bottom line [11], but there are different definitions for CSR.

According to the United Nations Industrial Development Organisation (UNIDO), CSR is a management concept that suggests the integration of social and environmental issues with a firm's operations and its interaction with the stakeholders [12]. Broadly, the term CSR reflects the concept of a triple bottom line approach whereby the businesses tend to achieve the balance in three core dimensions known as economic, environment, and social aspects while addressing the concerns of shareholders and stakeholders [11]. CSR is neither a charity nor philanthropy but purely a strategic management concept, and it is critical to understand this distinction. There are various benefits associated with it for the businesses that are actively implementing CSR in their operations, such as improved business reputation, improved quality and productivity, customer loyalty, access to the (new) market, and employee motivation [12]. The European Commission (EC) for enterprise and industry also has a similar definition of CSR; however, the commission emphasises taking ownership of the impact caused by individual business practices [13]. In academia, the term CSR is also quite broad; however, the researchers in recent years have focused on its impact on business and society, either positive or negative (Cruz and Boehe, 2008; [14]; Forsman-Hugg et al., 2013; Poetz et al., 2013). Academics as well as professional bodies describe CSR with similar definitions.

In modern history, Howard R. Bowen (1953) was among the initial scholars who defined CSR in a definition. According to Bowen, CSR is “the obligations of businessmen to pursue those policies, to make those decisions, or to follow those lines of action that are desirable in terms of the objectives and values of our society” (Bowen, 1953, p.6).

It was the first authentic definition of CSR in modern days which was published in the book *Social Responsibilities of the Businessman* by Howard Bowen in 1953 [15]. Bowen (1953) has raised the fundamental question in the book which is the basis of modern-day CSR, the question was “What responsibilities to society may businessmen reasonably be expected to assume?” A few years later, Heald (1957) further expands the definition; according to Heald, “CSR is recognition on the part of management of an obligation to the society it serves not only for maximum economic performance but for humane and constructive social policies as well” (Heald, 1957, p.375). Bowen (1953) and Heald (1957) have defined CSR through clear definitions. The literature on CSR further broadened in the 1960s and 70s, the emphasis remained on the relationship between business and society, and the responsibility of managers (decision-makers) to maintain the balance. Walton (1967) defined CSR as the following: “In short, the new concept of social responsibility recognizes the intimacy of the relationships between the corporation and society and realizes that such relationships must be kept in mind by top managers as the corporation and the related groups pursue their respective goals” (Walton, 1967, p. 18).

More or less, all of the above definitions provide a similar explanation of the phenomenon. There is a consensus on the obligations businesses have towards society. Most scholars are convinced that businesses have a responsibility to pay back to the society or community they operate in. CSR has emerged as a key strategic management issue in recent years (Moura-Leite and Padgett, 2011) and has become an integral part of every business organisation particularly in advanced countries. Also, there are no set boundaries for businesses, and instead, they can be innovative, and that is why the phenomenon has continued to expand and moving beyond businesses and into the supply chains.

### 2.2. CSR, Globalization, and Added Stakeholders

The International Standard Organisation (ISO) has introduced an updated definition for CSR which covers the broader stakeholders. According to ISO 26000, social responsibility is “the responsibility of an organization for the impacts of its decisions and activities on society and the environment, through transparent and ethical behaviour that contributes to sustainable development, including health and welfare of society, takes into account expectations of stakeholders, is in compliance with applicable law and consistent with international norms of behaviour and is integrated throughout and practiced in an organization's relationships” (ISO, 2010, p.3).

The proliferation of international trade and globalisation has impacted business strategies. There is greater awareness about the CSR practices related to labour conditions in supply chains along with the issue of human rights and environment protection (Kercher, 2007). Globalisation brings many new opportunities for businesses to access resources across the globe. Businesses capitalise not only through establishing supply chains and sourcing materials but also through outsourcing services to the countries with labour (Guttal, 2007). However, this phenomenon also brings challenges for businesses. Habisch et al. [16] assert that the debate about globalisation and sustainability has made CSR the most debated subject among politicians, consumer groups, trade unions, NGOs, and businessmen.

The current CSR debate is beyond multinational corporations and large firms to every business entity including small and medium enterprises (SMEs) [17]. The impact of globalisation has generated a new debate around the idea that businesses owe a responsibility to broader stakeholders (Kercher, 2007). This is a move away from the initial shareholder primacy which was defined by the famous economist Milton Friedman [18] where he argued that businesses' sole responsibility is to maximise the profit for its shareholders. The newer stakeholder idea is based on the combination of organisational management and business ethics (Freeman, 1984).

### 2.3. Supply Chain and CSR

The World Bank [19] has identified three types of supply chains that were used for global trade; they are known as

- vertical integrated chain (transnational company↔retailer)
- collaborative chain (producer/groups↔exporter↔importer↔retailer)
- transaction-oriented chain (multiple producers↔exporters↔importer↔retailer) [19]

The vertical chains are highly consolidated and closely connected as the retailers have direct contact with the producers. However, these kinds of chains are not common to buyers in developed countries and producers in developing countries [19]. The majority of the chains in developing countries were based on a transaction-oriented model where several producers (small-scale and large-scale) sell their commodities to the exporters; exporters then sell those commodities to the importers (Hobbs, 2010; Mondelaers and Huylenbroeck, 2008). This type of supply chains is complex as there is less communication and less awareness for the producers about the quality demands from the buyers [19].

A good example of this is the sports brand Nike in Pakistan, which is the major supplier for hand-stitched footballs for Nike with over 80% of overall production [20]. After the 1996 crisis, Nike suspended its ties with the Pakistani suppliers and overlooked its policies and codes. The brand refined the criteria and started business again with the Pakistani exporters, but this time to meet the CSR agenda was the top priority. The company explained the codes categorically, and to ensure the compliance, they established a local, regional office solely for this purpose to monitor the ethical standards (Khan and Lund-Thomsen, 2011). The football manufacturing industry of Pakistan is in the form of clusters based in the city of Sialkot and the suburbs. There are major factories, but due to huge demand, exporters have to rely on subcontractors who source from small factories and home stitching centres (Lund-Thomsen and Nadvi, 2010). Nike understands the complexity of this chain and goes beyond relying on the main contact by setting up a local, regional office. In this way, regular audits and checks can be conducted throughout the chain down to the home stitching centre to assure 100% compliance [20].

### 2.4. Global Value Chains

Developing country exporters are undergoing a transformation phase; the global requirement and codes of business conduct keep on changing. The element of ethics and the requirements of international certifications within the supply chains are mandatory now. Value chain governance is one way to ensure good food standards and trust relationships in the value chain (Vieira and Traill, 2008). A better understanding of the relation among the other actors in the supply chain can be of benefit to coordinators and agrofood business managers. Understanding the structure and building up the chain can also help in locating self-positioning, and this could lead to the development opportunities for individual actors across the value chain (Mikkola, 2008).

The global value chain (GVC) framework traces the complex links between vast scattered producers and global lead businesses. There is regular coordination of the processes between firms of worldwide distribution and production [8]. Though the GVCs are highly complex, it is unlikely that the main supplier or exporter has complete control over the entire value chain down to the bottom (Wiese and Toporowski, 2013). However, if local producers want to improve their position within global trade, there is an opportunity available by placing them into a GVC. There is always pressure from the leading firms on cost competitiveness and improved infrastructure. This pressure leaves the producer with two options: either inject more resources and investment or downgrade by squeezing the labour and working conditions. The latter is perhaps a short-term solution and will face difficulties over a period of time [8].

GVC is a mixture of opportunities and challenges particularly for businesses in developing countries as it provides an opportunity to access high-value markets in developed countries, as it is not simple to follow the procedures and requirements of the chain and also offers competitive prices at the same time (Sun and Zhang, 2009). GVCs are buyer-driven, and buyers are in a strong position to control the chain according to their needs, albeit it is not clear how they foster the system of upgrading in developing economies (Sun and Zhang, 2009).

### 2.5. Ethical Sourcing

Environmental concerns and sourcing of the products from other countries bring more challenges for the buyers. The situation led to the emergence of new terms such as reasonably sourced, fair trade, eco-friendly, and produced locally, used by the food retailers in advanced countries, in an effort to somewhat satisfy their customers and other pressure groups who were raising concerns about the poor working condition, child labour, and carbon emissions. The concerns were focused on imports from developing countries [21]. The buyers started to conduct interviews with suppliers, where questions have been asked about the ethical audits, and these interviews took place before the final decision (Pretious and Love, 2006). Governments use legislation to define their policies, but that legislation does not explain what is good or right; ethics plays the role of defining what is good and right. Without the element of ethical consideration, businesses would lose out in the longer term. At a time of globalisation and intense supply chains, it is critical to evaluate moral arguments in order to make judgements, and only by doing this, the organisations can come out with well-reasoned decisions. Failure to do so would cost the consumers' confidence (Manning et al., 2006). Although the major influence of price competitiveness, quality, and delivery time dominates the decision in selecting supplies from an international supplier, ethics audits do have an impact on this decision too (Pretious and Love, 2006).

### 2.6. Critique of Private Standards

Although different standards target various aspects and specific issues, they all have two particular core limitations, and they are, firstly, their inability to influence producers on how they put them into practice or how they enact them and, secondly, what role these standards play in rural development [22]. Some studies even found adverse impacts on the farmers in developing countries [10]. One more criticism that these private standards face is playing a part in allowing supermarkets to become such a powerful actor in the global value chains. As these standards are based on CSR-related activities, the supermarkets in the developed countries use them for profit maximisation through product differentiation (Sodano et al., 2008). The core reason behind the initiative of GlobalGAP was similar to the other quality assurance certificates, and that is to ensure their stakeholders that environmental and social responsibility standards are met throughout the production. The audit reports suggest that more attention has been given towards the production processes rather than on the environmental issues. Also, worker health and safety and welfare including a minimum wage rate are ranked as recommended and not as a major must [23].

A recent study on the effects of private standards on small-scale asparagus producers in Peru revealed the negative impact of these standards on small-scale farmers. It is argued that previous studies only concern about the certified farms and the financial gain but nobody considered the losses endured by small-scale asparagus producers in Peru. The country has a reputation in the international market for asparagus, and everyone who is involved in the production benefits from this. Since private standards came into practice, the balance has moved towards the large producers.

### 2.7. Motivation

Robbins and Coulter [24] defined motivation as a result of interaction between a situation and the person. It is a process to energise the efforts to achieve goals; they identified three characteristics of motivation which include energy, direction, and persistence. Energy reflects the intensity of effort made, direction guides to achieving a firm's goals, and persistence is to exert the effort until that goal is achieved. The duo also insisted on bringing compatibility between an individual's needs and organisation's goals for the best results.

It was argued by Honore (2009) that every business required motivated employees for that business or firm's survival; motivating employees could be as cheap as praising their efforts. Later, it was also acknowledged as a continuous process that has to keep on going (Goudas et al., 2011); motivation thus depends on the individual and the circumstances around that person (Strombeck, and Wakefield, 2008). It is certainly different than satisfaction (Mahesh and Kasturi, 2006), as it was previously argued by Pardee (1990) that motivation, job satisfaction, and reward systems are the components of one specific area of any organisation, where motivation has the strength to overlap the other two elements.

This theory was supported by Ramlall (2004), as he argued that the challenges of employee retention are faced by every small, medium, and large organisation regardless of their size and the role of motivation at work. He highlighted the importance of retaining critical and experienced employees as losing them would cost the organisation from an economic perspective, and it would also represent a loss of knowledge. Mango cultivation is greatly based on manual handling, and the process of water use, pesticide use, and taking the fruit from the tree requires certain expertise. All the workers at certified farms have been trained extensively to follow each step with care as the fruit is an exceedingly sensitive commodity. Also, these workers work on their own without supervision most of the time, as to cover a considerably large area; it is not possible to monitor each worker individually. Therefore, it is critical to retain such workers for sustainable performance of the particular farm. There are several theories associated with the concept of motivation; below are several pioneering theories, and most of the above definitions are somehow derived from these theories.

### 2.8. The Research Theoretical Framework

**Figure 1:**
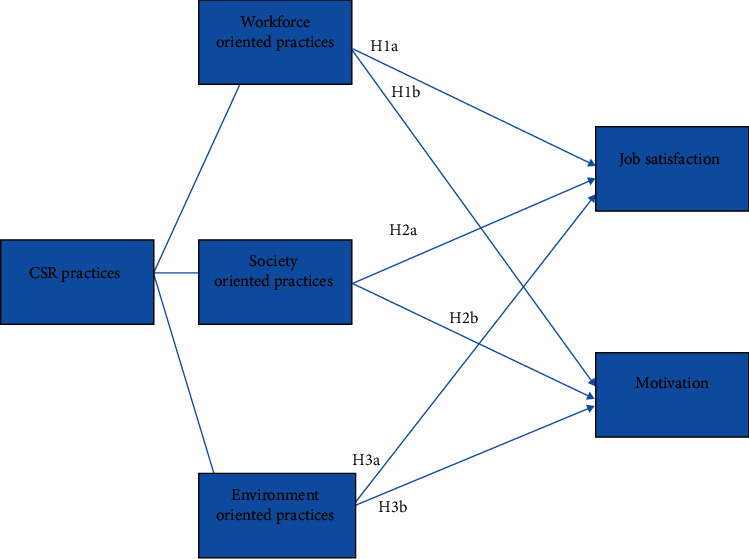
Proposed research model designed by authors. H1a: there is a positive relationship between workforce-oriented CSR practices and rural worker job satisfaction. H1b: there is a positive relationship between workforce-oriented CSR practices and rural workers' motivation. H2a: there is a positive relationship between society-oriented CSR practices and rural workers' job satisfaction. H2b: there is a positive relationship between society-oriented CSR practices and rural workers' motivation. H3a: there is a positive relationship between environmental-oriented CSR practices and rural workers' job satisfaction. H3b: there is a positive relationship between environmental-oriented CSR practices and rural workers' motivation.

The detailed literature survey led to the identification of the knowledge gap and the formation of the conceptual framework (Figure 1) to define and test the hypotheses. The literature review suggested that previous studies focused on macro (firm/farms/SMEs) level productivity (Leipziger, 2001; 2003; Rasche and Esser, 2006) in terms of financial gains (Kersting and Wollni, 2012) or access to the new markets ([17]; Sun and Zhang, 2009).

This particular study has attempted to explore the effects of CSR practices at the micro (individuals/workers) level. The hypothesis-based model to collect the quantitative data is inspired by a model used by [25] for research on CSR and SMEs in Uganda; also, the same model was previously used by the European Commission [17] for research on CSR and SME productivity in Europe. So, it is an established model to test CSR practices against the competitiveness of a firm.

The formation of the model (Figure 1) was in accordance with the population and sample under the subject. This study was held in Pakistan, and the researcher was convinced that the given model was best suited for the developing country context. The above model was used to assess the business performance of the SMEs, but this particular study is aimed at evaluating the farm workers' motivation, so the unit of analysis was different, and to adjust the model according to the purpose and the research questions, the researcher has amended the model accordingly.

### 2.9. Causal Relationship of the Variables

The World Business Council for Sustainable Development (WBCSD) discussed CSR at a summit held in 2001. The council defined CSR as the commitment of businesses to contribute to sustainable economic development, working with employees, their families, and the local communities (WBCSD, 2001). It was also argued by Clarkson (1995) and Waddock et al. (2002) that CSR stands on this fundamental idea of looking after a wider array of concerned stakeholders. Jamali and Mirshak (2007) discussed the scantiness of available empirical evidence on philosophy and practices of CSR in developing countries. In the same year, the European Commission for enterprise and industry directorate general's funded report—CSR and Competitiveness European SMEs' Good Practice (2007)—was published by KMU Forschung Austria, which is an Austrian Institute for SME Research. This research project found a positive relation between CSR practices and job satisfaction and employees' motivation. The study argued that the SMEs with higher involvement in CSR-related initiatives such as better and secure workplace, better society, and safer environment have improved their human and financial resources; the most prominent aspect was worker motivation, and job satisfaction had been improved that positively affect the employee retention, downfall in absenteeism, and better productivity. The same model has been tested by Turyakira et al. [25] where they conducted a similar study on the SMEs in Uganda; similarly, they found a positive link between CSR practices and job satisfaction. These studies inspired this particular research; however, it is different in terms of population and location of the research. Instead, all SMEs in this study have focused on certified mango farms, and only GlobalGAP certified farms were included in the study as it was the sole standard working with Pakistani mango growers. The standard has set criteria for CSR also known as codes of conduct. To establish a link between CSR practices and employees' motivation and satisfaction, the researcher designed this hypothesis model. Since this study was an attempt to look at CSR from a different perspective, with the element of private standards and its impact on workers and farmers, this model is the most appropriate. CSR is treated differently in developing countries, and western countries are trying to ensure compliance through private standards. The necessary attention was given to workers regarding their health and safety and wellbeing, because this was the first study of its kind in South Asia and especially in Pakistan. This model has also helped in identifying the factors that have the most influence on workers' job satisfaction and motivation and that will allow for improvement of the structure of existing standards. GlobalGAP does not address the social issues through its certification; the researcher decided to add them because issues such as child labour and women at work had a history in the Pakistani context, and even if there was no social consideration in GlobalGAP, such matters make an impact on the acquisition of a successful certificate.

The major dimensions of CSR are social, economic, and environmental [11]. However, they have been divided into many subdimensions. Forsman-Hugg et al. (2013) identified seven key dimensions of CSR that are related to the food chain. These include occupational welfare, product safety, environment, animal welfare and safety, nutritional responsibility, local market presence, and economic responsibility. This particular study was examining CSR in the context of GlobalGAP as a private standard and whether it has any influence on workers' motivation. The researcher chose workforce-oriented CSR, society-oriented CSR, and environmental-oriented CSR as the independent variables. Although the current mode of certificate does not include the society consideration, the researcher decided to add this into the conceptual model to see if there was any correlation.

The World Business Council for Sustainable Development (WBCSD, 2000) highlights the importance of employees by redefining CSR as a continuous commitment to behave ethically. Therefore, businesses are not only responsible for the economic contribution but also required to take care of their workforce and their families along with the community and society as a whole. It is vital to equip them with the training and skills to become even more productive in the coming years, and their retention is critical for business success. Robinson [9] found that workers perform their tasks in hazardous conditions, and they are not pleased with the situation, but due to limited resources and no job security, they have no choice. De Neve [26] discussed workers' prospects and highlighted two key factors that determine their choice of work derived first from their need for a livelihood and second from their sense of autonomy and dignity at work. He further argued that the sense of autonomy changes with time, and so do the livelihood needs. However, he found that livelihood needs took precedent after certain stages and dignity at work became less important.

The workforce is considered to be the backbone of any successful business, and when it comes to the agrofood industry, the importance of the workforce is increased as they are required to work on their own most of the time without any supervision. Workers associated with mango farming thus require considerable attention because mangoes are a delicate and highly perishable fruit that requires extra care and attention because their appearance has a strong influence on the consumer buying decision. A motivated and satisfied worker will be more productive and efficient as job satisfaction is linked to improved performance (Ramlall, 2004). Van Knippenberg [27] found a positive relation between work motivation and task performance.

In a case study of two separate firms that fully adopted and implemented CSR in their operations, Kim and Scullion [28] find a positive relationship between employees-centred CSR and work motivation. Rodrigo and Arenas (2010) identified three different types of employees' attitudes towards CSR implementation: dedicated employees, dissenting employees, and indifferent employees. De Neve [26] defined complying firms as those required to regulate workers' overtime, provide them with standardised contracts, and avoid contract and casual labour. The particular research in this study has focused on only those dimensions of CSR which are there to improve workers' working conditions and welfare rather than the overall impact.

Society responsibility reflects the local communities and society, how they are affected by an organisation's actions, and what businesses are doing to improve them. In developing countries, there are issues of child labour and woman empowerment. Uddin et al. (2008) argued the importance of healthy and stable communities and how the reputation as a producer and employer affects business competitiveness. Olejarova (2004) highlighted the importance of building a good relationship with neighbouring communities by minimising the damage to the environment because as soon as a business starts to work in any location it becomes part of that society. Public welfare services and voluntary work are the best examples of society-oriented CSR. Recruiting those who might struggle to get work otherwise is also categorised as a society-oriented CSR [17].

Environmental-oriented CSR represents the initiatives that had been taken to protect the environment by the businesses. There was a time when Carroll [15] produced his famous pyramid to explain CSR. The pyramid does not include environmental consideration, but today, the most prominent dimension of CSR is the environment protection.

Turyakira et al. [25] describe this as the measures taken by a business to minimise its harmful impact on the environment, for example, by using an environmentally friendly material, recycling, waste reduction, and pollution control. The FAO Report [29] explained the natural environment factors that occurred as a result of food production such as soil, water, genetics, and air. These factors have enormous impacts on society and environment in the form of climate change and disease, and it is then beyond the control of the producers. The report suggested that sustainable environmental practices could also become a source of value creation and competitive advantage.

The dependent variables for this study were job satisfaction and motivation; previously, it has been explained how different motivational theories work. As the literature identified that workers are the major stakeholders in developing countries when implementing any governance structure, the best possible outcome one can expect from this exercise is to have increased motivation among workers.

Johnston (2001) argued the company's strategy towards CSR and the employees and said that if a firm cannot assume a higher level of social responsibility towards its employees, it is unlikely to consider the other stakeholders including customers. The relationship between employees' attitude and CSR has never been explored in detail; a relative study had been carried out that found a positive relation between CSR practices and employees' job satisfaction (Tziner et al., 2011).

Lombart (2012) reported a rise in the number of employees that have been motivated through CSR implementation. The implementation process included the formation of employees' forums, arranging recreational activities and atmosphere, creating real rivalry and competition among the team, and arranging training and development courses for individuals. It was observed over the years that the numbers had been improved and the first outcome was a huge drop in absenteeism.

## 3. Material and Methods

### 3.1. Research Philosophies

The researcher's belief about the understudied social reality is critical in shaping the methodology they adopt. The ontological and epistemological positions sharpen the focus of the investigation and the chosen methodology. Blaikie (2007) explained this phenomenon as “The methodological perspectives are defined in terms of their ontology and epistemology, and include reference to the logic of theory construction, what counts as data, explanations and theory, criteria of validity, and views on the particular nature of social reality and the relationship between the natural and social sciences” (Blaikie, 2007, p.6).

Bryman and Bell (2007) described the concept of a research paradigm as a set of beliefs that control the ways in which research would be carried out and how the results will be interpreted. Johnson and Christensen (2005) argued that the research paradigm is a perspective that relies on shared values, concepts, assumptions, and practices. Crotty (1998) recognised the difficulties in drawing and distinguishing the concepts of ontology and epistemology. He argued that both concepts are related and mutually dependent on discussing the research issues and can lead to conflate each other. Scotland [30] suggested that the assumptions a researcher make are conjecture, so the underpinning of each paradigm is not possible to prove or disprove empirically. This research has discussed the ontology, epistemology, and pragmatism as the considered paradigms.

#### 3.1.1. Ontology

The ontology of this particular research is based on CSR compliance in the Pakistani mango industry and how it affects the rural workers. Burrell and Morgan [33] suggested that ontology contemplates assumptions about the essence of the phenomenon being investigated. This philosophical term portrays one's views about the nature of reality, and our assumptions or claims also reflect that perception too.

There are many possible ways to answer the question about the social reality; core assumptions about what exists, what factors it comprises, and what it looks like (Blaike, 2007). Stahl (2007) stated that the researcher needs to know what exists in order to investigate. It is the central point of any research regardless of the discipline (Klein et al., 1991). Guba and Lincoln (1989) argued that the researchers must consider the authenticity of ontological assumptions, as it would make it convenient to formulate more sophisticated and informed constructions. It would help the researcher and others in understanding the content and gain a sense of engaging with the constructions. The ontological assumption for this particular research was concerned with the rural workers' perception of good agricultural practices and their motivation. Guba and Lincoln [32] suggested that reality can be subjective and differ from person to person.

#### 3.1.2. Epistemology

The epistemological assumption for this particular research was to involve all stakeholders in a dialogue (interviews, focus groups, and questionnaires) to produce a meaningful analysis. Tsoukas and Chia (2002) attributed epistemology as a source to describe what we know and how we found it out. In other words, it can be described as a source which enhances existing knowledge. This study is classified as a social research, and according to Bryman and Bell (2007), the most fundamental purpose of social research is to find out about events that are happening in real life.

#### 3.1.3. Research Paradigm

The term research paradigm is also denoted as a research philosophy [31]. There are four components of a research paradigm, and they include ontology, epistemology, methodology, and methods [30]. However, Blaikie (2007) suggested that the initial process of defining the research philosophies (ontology and epistemology) leads to establish the research paradigm. The paradigms are those systematic sets that form our beliefs and methods that represent a distillation of what we think about the world (but cannot prove) [34]. According to Bryman and Bell (2007), the research paradigms are collections of beliefs that influence the researcher to conduct the research in a certain way and also how they interpret the results.

#### 3.1.4. Interpretivism

The interpretive attitudes propose that social reality is subjective. Interpretivism is an antipositivism stance (Crotty, 1998; Hatch and Cunliffe, 2006) that looks for culturally derived and historically situated interpretations of the social life-world (Crotty, 1998). In the social life-world, it is quite evident that individuals see things differently.

The context of this particular research is an impact assessment of socioeconomic and environmental practices through private standards in Pakistan. Interpretations of the individuals' responses comprised the background of this research. Saunders et al. [31] argued that individuals' responses can be extremely contextual and they cannot be generalised. In terms of epistemology, interpretivism is most closely associated with constructivism. Eriksson and Kovalainen [35] suggested that an interpretivist approach is closely related to the qualitative method techniques. They also acknowledged that this paradigm is hugely subjective in nature, and thus, its emphasis is on the language and the relationship between the researcher and the respondent.

#### 3.1.5. Realism

A common element between the interpretivist stance and the realist stance is the agreement on the fact that natural science and social science are different and that social reality is preinterpreted. In addition to this, the realists also consider that the science must be based on empirical data and is objective and rational. This position is in line with the positivist stance. Realism suggests that social objects can be explained scientifically, similarly to the quantitative analysis. This particular study also included quantitative analysis in order to explain the social objects and phenomenon. The positivists also claim the existence of a causal relationship, and these relationships provide a further basis for predictions. The realists argue that through observations, it is possible to understand the underlying mechanism, which is the case for qualitative analysis. Ultimately, realism considers aspects from both the interpretive and positivist positions.

#### 3.1.6. Pragmatism

Gray [36] argued that, in most of the cases, it would be necessary to mix qualitative and quantitative data in a single study where a pragmatist approach has been adopted. Saunders et al. [31] argued that if a researcher believes that choosing one position to conduct a particular research would be practically unrealistic, it would be appropriate to adopt the pragmatist position. It has allowed for adjusting methodology according to the merits of the research questions. In the context of this particular research, it was more appropriate to adapt the pragmatist approach as it was the most suitable paradigm to conduct this research. Also, Howe (1998) suggested that both qualitative and quantitative methods are compatible. He asserted that mixed methods are useful for building a good research design.

### 3.2. Research Approach

Research approaches are critical in defining the research design. Saunders et al. [31] suggested that it is vital to have a clear understanding of the chosen theory as it will decide the suitability of the research approach. There are two major research approaches, inductive and deductive. The deductive approach starts with a theory and hypotheses and then designs the appropriate strategy to collect data and then tests the proposed hypotheses. Conversely, the inductive approach starts with data and leads to building a theory [31]. The hypotheses and their relevance to the study draw a distinction between the adoptions of an inductive or deductive approach.

The deductive approach is largely used to test and validate the assumption or the proposed hypotheses, and the inductive approach usually produces a new theory that can be generalised [37]. This research started with a theoretical perspective, and that can be classified as a deductive approach.

#### 3.2.1. Integration of Inductive and Deductive Approaches

This research was carried out with a pragmatist position, and that position allows and involves combing the research approaches. The deductive and inductive approaches were integrated to produce a workable research. Both approaches are different to each other, and there are underlying differences between the two approaches. Blaikie (2007) stated that it is feasible to combine the two approaches. This particular research has successfully combined both strategies.  Table 1 highlights the differences between an inductive and deductive approach. Saunders et al. [31] made the distinctions in the approaches explicit in the given table by highlighting the factors which approach emphasises. According to them, the deductive approach involves the collection of quantitative data, and the induction approach adapts qualitative data to understanding the meanings and context better.

It has been argued in this study that there is very little data available on this particular issue of CSR compliance and rural workers' motivation. Only a few studies partially discussed this problem, but that does not represent the population being explored in this research. Lobe (2008) suggested that it is possible to conduct the qualitative phase first, and later, the result can be supported with quantitative data.

### 3.3. Research Design

Groenewald (2004) insists on choosing the right design for research that is in accordance with that particular study, that is, to pick the most appropriate design from the available options required in order to conduct a thorough study. Research design falls into two broad categories: experimental design and survey design; this study certainly falls under survey design. Further, there are subcategories of longitudinal design where data is collected from the same sample over the period of time at different points and cross-sectional design where data is collected at one single point (Ross, 2005). The data for this research was collected at a single point and thus falls under cross-sectional design.

According to Bryman and Bell (2007; 2011), a cross-sectional research design comprises a collection of data at a single point from more than one case through testing the connection between different variables. Further examination of such data then establishes the patterns and associations. The design is also known as social survey design where researchers are interested in testing variation among people, organisations, or nations.

### 3.4. Mixed Methodology

There is a significant increase in the number of researchers who are turning to mixed method techniques of research to make the result more powerful and to widen the scope of their studies (Johnson and Onwuegbuzie, 2004). However, with this rise, there is confusion among them too on how to combine the quantitative and qualitative research best in order to achieve conclusive results or findings. This method is considered dynamic in terms of generating the combinations for sampling and data collection. The researcher positioning and view decide the nature of the combination and what techniques will be used to perform that as well as how and where he wants to mix stories and numbers or keep them separate throughout and conclude separately [38].

Johnson and Onwuegbuzie (2004) explain the usefulness of mixed methodology; they argued that mixed method research is one of three research paradigms which are a qualitative, quantitative, and mixed method. They define mixed method research as a method where researchers combine or mix qualitative and quantitative data in order to achieve a detailed analysis; it is an attempt to provide researchers with more freedom to choose how they want to conduct a study according to the needs of the study.

**Figure 2:**
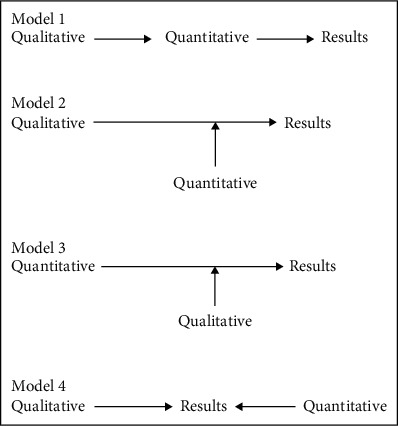
Mixed research model.

Steckler et al. (1994) suggested four models (Figure 2) of methodological triangulations. The first model uses multiple measures to investigate the same phenomenon. The second model in Figure 2 involves the observation of different fields. The third model involves theoretical triangulation that involves multiple theories. The fourth model involves methodological triangulation which considers the equal use of the qualitative and quantitative methods.

This particular study has adopted the second model by using multiple measures to explore the same phenomenon. The purpose was to establish the usefulness of private standards for ensuring compliance with CSR practices and if those practices affect workers' motivation (Figure 2). The study draws on qualitative data and analysis, and all four research questions have been answered with qualitative analysis. However, the findings of the core research question which is about the relationship between CSR practices and workers' motivation were further investigated and supported by quantitative data's findings.

The most fundamental thing in a research project is the research question, and the methodology should be flexible enough to address the core question(s). It is also critical for the researcher that they can best use a mixed method research, and one essential step is to consider all the relevant major characteristics of both the qualitative and quantitative methods. It will allow the researcher to highlight the strengths and weaknesses of both methodologies in context with their study (Johnson and Onwuegbuzie, 2004).

Mixed method research provides a platform to reinforce the trust among communities as there is an awareness of the power of both quantitative and qualitative data (Mertens, 2007). Johnson et al. (2007) argued the stages of research and discuss what could be the best stage at which to mix the methods. Mixed data requires some integration, and researchers have to be careful when choosing the philosophical position that best suits a mixed method research. The pragmatism philosophy is the most popular among the advocates of mixed methodological research as pragmatism provides epistemological justification and logic for choosing this method. Research paradigm employing pragmatism as a philosophy is anchored on the usage of mix methods in a situation where it is leading to a nexus of occurrence in the context of one's research questions.

#### 3.4.1. Justification for the Choice of Mix Methods

Over the last century, there has been a debate about qualitative and quantitative research methods, and advocates for both paradigms claim that certain methods are better. There is a list of strengths and weaknesses for both methodologies [34, 39]. A comparatively new pragmatism of mixed methodology certainly causes a great deal of noise in academia, and researchers from the qualitative and quantitative schools of thoughts discussed whether a study can build a consensus by adopting both methods at the same time when these both methods are opposite to each other. However, many researchers have successfully adopted this new paradigm and draw more definitive results (Johnson and Onwuegbuzie, 2004).

The nature of the study was the core reason to choose mixed methodology for this research, since this was an ex-postimpact assessment research about GlobalGAP as a private standard in the Pakistani mango industry, and the researcher had tried to investigate the impact on both, the mango farm owners and farm workers. There were about thirty-one certified mango farms in Pakistan; the numbers of farms were not enough to conduct a quantitative study, and also, the nature of the research questions cannot be answered to the satisfactory level through the questionnaire survey alone. The subject and population of this research have never been examined before, so thorough understanding and knowledge were required in order to understand why growers were acquiring the GlobalGAP certificate since it was a requirement by the supermarkets in developed country and Pakistani mangoes only sold at the community markets. To examine the potential impacts on the motivation of workers, a quantitative study was required as all the certified farms have a minimum of approximately fifty workers each and the population was large enough to conduct a statistical analysis.

The findings from both methods made a more conclusive result (Johnson and Onwuegbuzie, 2004) and had allowed for drawing a solid verdict about the usefulness of GlobalGAP in the Pakistani mango industry. The results are broad enough to open new directions for further studies as the available literature is still unclear over the role of private standards in Asia, particularly in Pakistan. The proposed research method was carefully selected and integrated best with the core research questions. It was the first of its kind of research among the chosen population, so it was vital to have complete in-depth analysis for better understanding.

### 3.5. Instrument Development and Case Selection

This research was an impact assessment of private standard adoption practice to comply with CSR requirements and its effects, as the chosen population was the Pakistani mango industry, and there was only one major private standard working with the growers and exporters in Pakistan, and that was GlobalGAP. Therefore, this study was based the GlobalGAP certification and its impact on the Pakistani mango industry.

The researcher tried to test the relationship between CSR practices at certified mango farms and workers' job satisfaction and motivation. It was the first study conducted in Pakistan to analyse the impact of the GlobalGAP, and that required a thorough analysis based on sound data. In order to collect comprehensive data, the researcher decided to adopt mixed methodology that was based on both quantitative and qualitative data. A quantitative questionnaire survey could have served the purpose of testing the workers' motivation, but that might look meaningless without the input from the farms' owners. CSR is still in its developing phase in Pakistan, and as this study was conducted in rural Punjab, it was unlikely that the concerned stakeholders understand the importance of this and how it could change in the future. The motives behind the adoption of the GlobalGAP in Pakistan were not known. This, alongside knowledge of the extent of what it had achieved so far and what represented its barriers, all needed to be known for a comprehensive impact assessment and analysis.

The questionnaire for quantitative data was designed in line with the conceptual framework and the research hypothesis. The independent variables were derived from the literature review of agrofood private standards and CSR practices; the three most important elements of CSR regarding private standards are workforce, society, and the environment, even though GlobalGAP is not particularly keen on society issues and focuses more towards traceability. The researcher added the society element to test how stakeholders would react to it, and the findings could help in shaping the standard in the future.

Pollard (1985) openly confessed that personal contacts and circumstances influenced the decision to choose the particular location as a case study. Also, Hammersley and Atkinson (1995) suggested that a close consideration must be given to the suitability and feasibility of the chosen location. The Punjab province has two major mango-producing clusters, situated in Southern Punjab known as Multan and Rahim Yar Khan Regions. Personal background and local knowledge helped the researcher during the field trips for data collection. The trips were conducted between August 2015 and April 2016.

### 3.6. Data Collection

Mangoes start to arrive on the market from May to August, so the timing of the field trips was ideal for observation of the complete harvesting procedures including postharvesting procedures. Mangoes are a once annually commodity, and unlike other crops, mango trees require less maintenance and are usually self-sufficient. Almost all the mango farms have temporary staff of around 25 to 40 workers depending on the size of the farm. During the season, there are temporary workers between 250 and 400 per farm.

The field trips were conducted during the high season; the quantitative data was collected from all the workers who were willingly available to participate in the study. In the questionnaire, they were asked to confirm if they were permanent or temporary seasonal workers, and that in fact helped in adding another dimension to the findings as to how preferences vary among permanent and seasonal workers. Also, it was in the best interest of the farm if it could attract temporary workers back in the following seasons as they were trained to work according to the high-quality requirements.

### 3.7. Data Analysis (Tools and Techniques)

The data from questionnaire survey answers were transferred to an MS Excel sheet prior to uploading the data onto SPSS. That data was then analysed extensively from different angles and methods. The qualitative data presented an even tougher challenge, not only because of its nature and sensitivity but also because multimethods such as linear regression and multivariate regression were adopted, and to organise all the information consumed a great deal of time during the analysis phase. A coding technique was utilised for the interoperation of the interviews, observation, and other documentary evidence to pick the key points and similarities.

#### 3.7.1. Validity

During the field trips, a total number of 17 certified mango farms were visited in the Punjab province, and that was the total number of the certified farms in that region. Therefore, it is safe to say that the study has covered the entire population in the area. Altogether, twenty-one (21) individual interviews were conducted with the growers, exporters, and other key players, along with the seven focus group interviews. For the quantitative data, the researcher had 349 valid, usable questionnaires filled in by the employees and workers at mango farms. A total number of 400 forms were distributed among the workers. The data was significant enough to generalise the findings. Although the questionnaire survey and interviews do not represent the entire mango industry, the data was sufficient enough to ascertain the findings, and that reflects a general understanding about the GlobalGAP among the stakeholders in the Pakistani mango industry.

#### 3.7.2. Research Setting and Sample

This research was carried out in the Punjab province of Pakistan; the population for the study consisted of GlobalGAP certified mango growers, farm owners, farmers, on-farm workers, exporters, and NGOs. For the qualitative data, the researcher conducted interviews with the farm owners or the top executives at each certified farm and exporters. USAID was the main donor and helped every farm in arranging finances required to acquire the certificate; the researcher interviewed the CNFA (Cultivating New Frontiers in Agriculture) officials who also represented USAID in Pakistan. The provincial government of Punjab initiated a research centre specialised for mangoes in the city of Multan, and few officials were interviewed from that research centre too. Altogether, there were 21 individual interviews, and seven focus group interviews were conducted throughout, for the qualitative data.

In addition, an email interview was also conducted with Professor Ray Collins from the University of Queensland, Australia. Professor Collins and his team have been working with Pakistani mango growers under the Agriculture Sector Linkage Program (ASLP) since early 2006. They had also published a report on the Pakistani mango supply chain constraints (2006) and a journal article on how to make marketing a tool for the sustainable supply chain (Collins and Iqbal, 2011). These were the only established published works which were available on Pakistani mangoes in terms of supply chain improvements when this particular research was initiated. It was critical to establish an understanding of the ongoing projects which Professor Collin was leading and to seek his views about the developments and progress of the mango industry.

Furthermore, the researcher's observation and documentary evidence were also integrated into the qualitative data. The observations were noted down to consider compliance with physical requirements of GlobalGAP such as clean toilets, staff room, secure and separate room to keep all the chemicals and pesticides, protective clothes (apron, gloves, and masks), secure equipment, and wastage management. Documentary evidence was based on record keeping and the information displayed. GlobalGAP has compulsory major must criteria for complete documentation of all the activities on the farm. The growers are required to maintain the record of pesticides and fertilizers that have been used, their quantities, date, and authorisations. To establish compliance, it was critical to view and observe these pieces of evidence.

For the quantitative data, the population consisted of farm workers and on-site workers at the certified farm. Since the certified farms must have all the facilities in-house, including storage and packaging units, they were expected to have much more labour than the noncertified farms. The researcher expected around 300-400 site workers to participate in this research and was able to collect 374 filled questionnaires out of 400 distributed questionnaires. A questionnaire translated into Urdu (the local, national language) was requested to be filled in by the voluntary participants; the researcher administered this process. The survey was conducted during the field trips to Pakistan during harvesting and post-/preharvesting season.

## 4. Data Analysis and Findings

### 4.1. Research Sample

As the study is based on GlobalGAP certified mango farms in Pakistan, the sample consists of workers at the certified farms only. Around 400 questionnaires were distributed among the farm workers (managerial and tactical employees), to collect the required information. Out of 400 questionnaires, a total of 374 were completed. That represents a useable response rate of 93.5%. However, only 349 of them were found to be useful as the rest of them did not pass the criteria to be literate enough to understand the questions. Two initial questions were designed to test the literacy level of the participants. The numbers were further cropped to 316 as the responses with 0.00, standard deviation (SD) were eliminated. 0.00 SD means no variance in the answer, for example, if the respondent ticked all 1s or 2s. There is an exclusion for only one 0.00 SD (Table 2), and that is for the gender data; all the participants were male, and that hugely reflects the gender inequality; however, the causes and excuses have been discussed in the previous chapter. To test for adequacy of the sample, the Kaiser-Meyer-Olkin (KMO) test was conducted with SPSS. According to Rennie (2002), the value closest to 1 represented a good fit.  Table 3 shows the KMO as calculated at 0.80. It was found to be significant at *P* < 0.001, and it confirmed that data was factor analysable.  Table 1 shows communalities to confirm that the KMO was calculated for all 26 items mentioned in Table 4.

The questionnaire was based on Likert scale style questions in Table 5, where respondents were given choices to choose from strongly agree to strongly disagree, and each response has been assigned a number from 5 to 1 to feed the answer into the SPSS.

There was also a demographic section in the questionnaire where respondents were asked about their age, experience, qualification, etc.

### 4.2. Descriptive Analysis

The study uses five variables: three independent variables named as workforce-oriented practices, society-oriented practice, and environment-oriented practices and the two dependent variables named as job satisfaction and motivation. As the data was collected through a Likert scale style questionnaire, all the variables were divided into items in order to collect the relevant information. Eight items represented workforce-oriented practices named as WFOP1 to WFOP8, four items represented society-oriented practices named as SOP1 to SOP4, four items represented environment-oriented practices named as EOP1 to EOP4, four items represented job satisfaction named as JS1 to JS4, and six items representing motivation named as M1 to M6. This study's findings were incredibly consistent with the literature; however, there were some significant contradictions that have also been identified, which will be discussed in the following chapter. There was a significantly high mean score for the majority of the responses which reflect a strong agreement with the given statements.

The environmental-oriented practices (EOP) were examined, and there were four items added to the questionnaire seeking responses on the key environmental practices which were in line with the requirements of the GlobalGAP. The highest mean value of 4.88 was found for EOP1 which was use of pesticides and fertilizers. GlobalGAP has a major must clause for the use of chemicals, and the mean value for EOP1 confirms a strong compliance as the majority of the respondents strongly agreed that their respective farm only uses the chemicals as advised by experts and that these were also secured in locked storage.

The item EOP4 which was water management fetched the second highest mean value of 4.79. EOP3 which was recycling attracted the mean value of 4.78, and EOP2 which was waste management also had the higher mean value of 4.67. The SD values (*σX*) for all 4 items also remained low as the highest value was found for EOP2 at 0.579. Unsurprisingly, there was no disagreement on the environmental issues, and all the participating farms were found to be in full compliance with the GlobalGAP requirements. The obvious reason was the pressure from the certifying body as environmental issues have a major must clause, and moreover, enormous efforts have been input by the growers with the help of USAID to ensure that they met the critical criteria for environmental standards.

### 4.3. Data Reliability

Prior to conducting the correlation and regression analysis, it was critical to establish if the data was reliable enough to produce correct results. The data reliability was analysed using Cronbach's alpha test with the help of SPSS. The alpha closer to 1 shows a perfect fit. Cronbach's alpha calculates the internal consistency of the data. Most of the studies suggested that an acceptable level of alpha is a minimum of 0.7 (Lance et al., 2006), but later, it was argued by Suhr and Shay (2009) that for academic research purposes the alpha is significant at 0.6 and must be acceptable. The overall alpha in Table 6 shows 0.924 which is significant given that the higher the value of alpha, the stronger the internal consistency between the variables.  Table 7 provides the details about the scale of the item(s) deleted. So, if SOP2 was removed from the analysis, the alpha would have increased to 0.927 as mentioned in Table 8.

The alpha for independent and dependent variables has also been tested separately. Tables 7 and 9 show the state of internal consistency between the items for the independent variables.

Table 7 shows Cronbach's alpha for the independent variables. All the independent variables have been tested in combination as they all reflect on the same phenomenon and that is CSR practices.

The alpha was found at 0.822 which is significant in Table 9. The high value for alpha confirms that there was significant consistency between all the observed independent variables. Below are the alpha calculations for the dependent variables.

Table 10 shows the alpha as 0.677 for the first dependent variable that is job satisfaction.  Table 11 shows alpha if certain item is deleted. If JS4 was excluded from the analysis, the alpha would have increased to 0.714. However, the analysis was carried out with all four items, as the overall alpha was >0.6 in Table 12.

Table 13 shows alpha as 0.911 for the second dependent variable that is motivation.  Table 14 shows alpha if a certain item is deleted. The alpha shows significant internal consistency between the items chosen to access motivation.

### 4.4. Combined Value Model

As the statistical model required to be tested with different tests and combinations, the relevant items were computed to have the data under the actual variables. The compute variable function in SPSS was used. The entire workforce-related (WFOP1 to WFOP8) items were added to create the variables workforce-oriented CSR, society-oriented CSR, and environment-oriented CSR. For the dependent variables, the items have been grouped into two variables and that were job satisfaction and motivation mentioned in Table 14.

### 4.5. Correlation

As the data was collected through a Likert scale style questionnaire and the findings were presented in categories in the ordinal form, Spearman's rank correlation coefficient (*r*_s_) was deployed to assess the correlation between the variables. According to the data, the two dependent variables job satisfaction and motivation themselves shared a strong correlation at *P* = 0.740. This particular finding was significant at *P* value 0.000 which is *P* ≤ 0.01. There was a strong correlation between the workforce-oriented practices and the job satisfaction at *P* = 0.671 and an even stronger correlation between the workforce-oriented practices and the motivation at *P* = 0.710. The result proves the first two hypotheses which were the following:

(H1a) There is a positive relationship between workforce-oriented CSR practices and rural workers' job satisfaction

(H1b) There is a positive relationship between workforce-oriented CSR practices and rural workers' motivation

The correlation was also significant at the *P* value 0.000 which is *P* ≤ 0.01 for both the above hypotheses. Among the main variables, particular items were strongly correlated with each other. Namely, WFOP5 (forced overtime) has a strong correlation with JS2 (work-life balance) and M3 (equal opportunities) at *P* = 0.671 and *P* = 0.662, respectively. The majority of respondents have shown strong agreement with no forced labour with the mean value at 4.28, resulting in more respondents expressing that there is a balance between their work and home life.

Another interesting relation has emerged between WFOP8 (performance-based rewards) and M3 where the correlation was strong at *P* = 0.606, so the study established a strong positive relation between performance-based rewards and equal opportunities at work. There was one negative, weak correlation found between WFOP7 (training) and JS1 (job security) at *P* = −0.18; however, it was not significant at *P* value 0.750 which is *P* ≥ 0.05.

There was moderate positive correlation recorded between the society-oriented practices and job satisfaction at *P* = 0.476 and between SOP and motivation at *P* = 0.489. These results validate the third and the fourth hypotheses which were the following:

(H2a) There is a positive relationship between society-oriented CSR practices and rural workers' job satisfaction

(H2b) There is a positive relationship between society-oriented CSR practices and rural workers' motivation

For the above hypotheses, the correlation was significant at the *P* value 0.000 which is *P* ≤ 0.01.

The most prominent correlation was found between the items SOP4 (child labour) and M3 (equal opportunities) at *P* = 0.617. The values suggest that the respondents who showed strong agreement with the fact that their farm has a clear policy about child labour attracted a mean value of 4.59, also showing a strong agreement with the fact that there were equal opportunities at their workplace. Another significant correlation has been found between SOP4 and JS3 (fair treatment) at *P* = 0.571, which shows the relation between the elimination of child labour and the fair treatment at work.

For the third independent variable which was the environmental-oriented practices (EOP), there was a moderate positive correlation found between EOP and JS at *P* = 0.530 and between EOP and motivation at *P* = 0.463; both values were considered significant at the *P* value 0.000 which is *P* ≤ 0.01. These results validated the fifth and the sixth hypotheses, which were the following:

(H3a) There is a positive relationship between environmental-oriented CSR practices and rural workers' job satisfaction

(H3b) There is a positive relationship between environmental-oriented CSR practices and rural workers' motivation

The most prominent correlation found among the items was between EOP1 (pesticides and fertilizers) and JS4 (working conditions) at *P* = 0.614. This finding is particularly significant for this study as the respondents correlate the improvements in the use and handling of the pesticides and fertilizers with the better working conditions.

It was also consistent with what the study found during the focus group interviews with the workers.

Spearman rho's correlation suggests that there is a positive relation between CSR practices and the workers' job satisfaction and motivation. These findings were also tested significantly against the *P* values at *P* ≤ 0.01. Apart from the combined relationship between the independent and the dependent variables, there were also some interesting relationships which have been discovered among the individual items within those variables. However, in this section, only the magnitude of the correlation coefficient has been examined, and to identify the causation, a more rigorous technique of multivariate regression analysis was adopted to explore the causal relationship between the variables.

### 4.6. Regression (Linear)

Spearman rho's correlation has identified a significant positive correlation among the variables. However, to make a prediction about the dependent variables (job satisfaction and motivation) on the basis of the independent variables (workforce-oriented practice, society-oriented practice, and environment-oriented practices), a linear regression equation was tested to make the prediction. It was a step towards testing the independent variables simultaneously against the dependent variables through multivariate regression, which is discussed further in this chapter.

### 4.7. Multivariate Regression

The Multiple Regression Correlation (MRC) is a technique often used when testing the relationship between multiple independent variables and a single dependent variable. There were two dependent variables in this study, so they have been tested separately in two MRC models, first the relationship between WFOP, SOP, EOP, and job satisfaction and then between WFOP, SOP, EOP, and motivation. At the first stage, a simple linear regression model was run to test the correlation among the variables separately; during this phase, a one-to-one regression coefficient (*r*^2^) was calculated to test the relationship.

One of the objectives of this study was to identify CSR practices that have the most influence on the job satisfaction and motivation of farm workers. A stepwise procedure was adopted to analyse this MRC model with the help of SPSS20; this procedure not only eliminates the nonsignificant variable(s) but also ranked the independent variables in order to understand the most prominent predictor (Hu and Ansell, 2007).

### 4.8. CSR Practices and Job Satisfaction

The stepwise procedure excluded the SOP from the calculation and predicted job satisfaction only on the basis of WFOP and EOP. There was a significant positive relation between CSR practices (WFOP and EOP) and job satisfaction at adj *r*^²^ = 0.634; this was also significant at *P* value 0.000 which was *P* ≤ 0.01. It transpired that job satisfaction is dependent on WFOP (*t*‐value = 18.107) and EOP (*t*‐value = 6.720); both the *t*-values were also found significant at *P* ≤ 0.01. The SOP counted for the *t*‐value = 1.242, but this was not significant as *P* value was found at 0.215, which has crossed the borderline of *P* ≤ 0.05.

Regression equation:

(1) $$\begin{array}{c} \text{Job satisfaction}=\beta 0+\beta 1=\text{workforce}‐\text{oriented practices}+\beta 2=\text{environment}‐\text{oriented practices}. \end{array}$$

The standardised coefficient beta values ranked the variables as the best predictors; the beta for WFOP was found 0.669 which was higher than the beta for EOP which was 0.248. On the basis of the beta value, it can be concluded that the workforce-oriented practices were found as the best variable to predict the job satisfaction.

### 4.9. CSR Practices and Motivation

There was a significant positive relationship between CSR practices (WFOP, SOP, and EOP) and the motivation at adj *r*^2^ = 0.571; the relationship was significant at *P* ≤ 0.01. It explained that motivation is dependent on the WFOP (*t*‐value = 14.712) with *P* value 0.000, the EOP (*t*‐value = 3.003) with *P* value 0.003, and the SOP (*t*‐value = 2.076) with *P* value 0.039; all the relevant *P* values were found significant at *P* ≤ 0.05.

Regression equation:

(2) $$\begin{array}{c} \text{Motivation}=\beta 0+\beta 1=\text{workforce}‐\text{oriented practices}+\beta 2=\text{society}‐\text{oriented practices}+\beta 3=\text{environment}‐\text{oriented practices}. \end{array}$$

The WFOP beta was found at 0.638, the beta for SOP was found at 0.097, and the beta for the EOP was found at 0.132. The beta ranked index showed that the WFOP was the top predictor to predict the motivation; the second-best predictor was found as the EOP; again, the SOP was least significant in terms of predicting motivation. These findings were significant in many ways. The GlobalGAP major must considerations were found towards the environmental-oriented practices, and the workforce-oriented practices ranked as a minor must, but according to this study, the workforce-oriented practices have the greatest influence on the workers' job satisfaction and motivation. Interestingly, society-oriented practices were not currently considered a requirement from the GlobalGAP, but that even have some significant influence on worker motivation.

## 5. Conclusion and Discussion

### 5.1. Reflecting on the Research Questions

(Q1) What is the relationship between CSR practices and workers' job satisfaction and motivation?

This question was given the central place in this study as the available literature is underdeveloped and insufficient on this particular issue. It was proposed prior to conducting this study that there is a positive relationship between CSR practices and the workers' job satisfaction and motivation. The findings are in line with the proposed hypotheses. The results from both the qualitative and quantitative analyses confirm that the workers at the certified mango farms were found to be extremely satisfied with the facilities and training they received, and they also expressed their desire to contribute more to the success of their respective farms. The main influence on the growers is the healthy competition within the country, and every grower's desire is to sell their mangoes at the premium supermarkets. The growers also acknowledge that their workers are more trained and aware and that they are capable of making their own decision.

Mango is a yearly crop, and every grower relies on the workforce to be available at the right time. They look after the workers in a way which means they continue to stay with them and also the temporary workers, so they come back next season to work with them. The reaction from the workers was encouraging; the conditions have been improved, but they are not perfect yet. However, even the small initiatives by the growers are popular among the workers. The traditional terms of working and nonregular income have always been challenging disadvantages for the labour market in the Pakistani agriculture industry especially for those who work in the rural areas.

Good agricultural practices ensure a regular income at the national minimum wage rate. This was praised as being the most beneficial feature for the poor workers. It brings some sustainability into their daily life. They can budget for their needs, and they have more control over their finances now. The other facilities such as sheds, common rooms, toilet, and protective gears were also highly regarded by the workers. After the regular income, the workers were found to be enthusiastic about the training they received at the certified farms, the training empowers them, and they can work on the terms which are suitable for them with the mutual consent of the management.

(Q2) What role does the GlobalGAP certification play to ensure CSR compliance at certified mango farms in Pakistan?

The second research question was proposed to verify whether CSR compliance was achievable through a private governance structure. Although the GlobalGAP is not particularly a social standard, it does cover some of the most critical issues that come under the CSR umbrella. The issue of environmental protection, child labour, worker welfare, health and safety, and record keeping are all covered in GlobalGAP certificates, and none of them were being practised by the mango growers in Pakistan prior to the adoption of the standard.

The growers have raised several concerns about the audit process that ensures compliance. However, at the same time, they also acknowledge the gradual improvements in the system. The key role GlobalGAP played in ensuring the compliance was the initial upgrade process that involves structural changes at the farms, the building of modern pack houses, and most importantly the training and education of growers and workers. Despite the weaknesses in the existing audit system, the growers were found to be enthusiastic about the good agriculture practices.

The focus group interviews with workers and the questionnaire survey data revealed that the farms' management were complying with the requirements of GlobalGAP. However, there is a need to improve the audit system in Pakistan. There are few audit firms currently operating in the region, and it was unclear if they have the capacity to meet the requirements. GlobalGAP must address this issue by motivating and inviting more professionals to join, and this can be done through induction seminars and training workshops.

(Q3) What difference does the GlobalGAP certification make for the income, work, and environmental conditions of mango farmers and on-farm workers in Pakistan?

The third question is aimed at identifying what difference the GlobalGAP made for socioenvironmental conditions of the workers. Pakistan is a developing country, and although it is an agricultural country, the agriculture sector requires huge reforms to protect the growers and the workers in particular. With little support from the government and limited resources, the facilities the certified growers are offering to their workers are commendable. The GlobalGAP certainly has huge impact to uplift the worker socioenvironmental and working conditions. The workers do acknowledge these initiatives and were found to be more committed towards their personal growth and the growth of their associated farms.

Health and safety of the rural workers always remained a question for the policymakers. The certified farms' workers were provided with the latest cutters, and they have been also been equipped with the modern training on how to safely use and secure those cutters. A worker was jubilant during a focus group, saying that he was no longer required to climb the trees. This practice works for the growers as it certainly reduced the losses and for the workers too as it prevents them from sustaining life-threatening injuries.

The issue of wages is still not streamed line for the workers who work in the agriculture. Most workers are paid after the crop, and some are even paid with rice and wheat instead of money. The GlobalGAP does not promise higher wages but ensures the payment of a minimum wage on time. It was observed during the field trip that workers were happy as they received regular income, some complained that it should be higher, some suggested annual bonuses, but there was a large consensus that they get their salary on time mostly on a monthly basis. Some workers even said during the data collection that they get better wages as compared to their previous farm which was not certified.

(Q4) Does the adoption of standards such as the GlobalGAP present a challenge for the mango growers in Pakistan?

The fourth and final research question seeks to analyse the challenges faced by the mango growers in Pakistan who have adopted the GlobalGAP. The data analysis and discussion chapters highlighted several benefits and opportunities for the participating mango growers in Pakistan. Also, the interested growers are supported by USAID and the provisional government to meet the financial requirements. Despite all of this, the findings of this study are consistent with Hansen and Trifkovic [40], that the standard is most suitable for middle- to large-scale growers. The small-scale producers cannot cope with the required volume and the sustainable supplies, as these are the major requirements from the supermarkets in the advanced countries. However, the GlobalGAP does provide the options for the small-scale growers to form a group or consortium to apply for a group registration under option 2 of the producers' groups. There are two active groups of mango growers working in Pakistan, one is in Sindh cluster and the other operates in the Punjab.

There was a small fraction of producers who initially acquired the GlobalGAP but failed in establishing links with foreign supermarkets. Those growers think that the renewal cost is a burden as they are dealing in different markets which are not strict about the standards. Some of them even cancelled their ongoing subscription, but they are still benefiting from the learning they have learnt through the GlobalGAP program.

The linear regression analysis shows that there was a significant positive relationship between WFOP and job satisfaction at adj *r*^²^ = 0.582 with *P* value 0.000 which is *P* ≤ 0.01. It transpired that job satisfaction was dependent on WFOP (*t*‐value = 20.97), and this value was also significant at *P* ≤ 0.01, with a 99% confidence level.

Regression equation:

(3) $$\begin{array}{c} \left(\text{H}1\right)\text{ Job satisfaction}=\beta 0+\beta 1=\text{workforce}‐\text{oriented practices}. \end{array}$$

The second hypothesis also validated that there was a significant positive relationship between WFOP and motivation (adj *r*^2^ = 0.543) with a significant index of *P* ≤ 0.01. The numbers suggested that motivation was dependent on WFOP (*t*‐value = 19.37); this value was also found significant at *P* ≤ 0.01 with a 99% confidence level.

Regression equation:

(4) $$\begin{array}{c} \left(\text{H}1\text{a}\right)\text{ Motivate}=\beta 0+\beta 1=\text{workforce}‐\text{oriented practices}. \end{array}$$

The results from the regression model to test the relationship between SOP and JS show that there was a significant positive relationship between SOP and JS (adj *r*^2^ = 0.249), so the JS is dependent on SOP with *t*‐value = 10.26; both the regression coefficient and the *t*-value were significant at *P* ≤ 0.01. These numbers validated the third hypothesis.

Regression equation:

(5) $$\begin{array}{c} \left(\text{H}2\right)\text{ Job satisfaction}=\beta 0+\beta 1=\text{society}‐\text{oriented practices}. \end{array}$$

There was a significant positive relationship found between SOP and motivation at adj *r*^2^ = 0.237, which shows that motivation is dependent on SOP with *t*‐value = 9.941. The coefficient and regression both were considered significant at a *P* value of 0.000 which was *P* ≤ 0.01.

Regression equation:

(6) $$\begin{array}{c} \left(\text{H}2\text{a}\right)\text{ Motivation}=\beta 0+\beta 1=\text{society}‐\text{oriented practices}. \end{array}$$

A significant positive relationship was found between EOP and JS at adj *r*^²^ = 0.253; it shows that job satisfaction was dependent on EOP (*t*‐value = 10.365); both values were significant at *P* value 0.000 which was *P* ≤ 0.01.

Regression equation:

(7) $$\begin{array}{c} \left(\text{H}3\right)\text{ Job satisfaction}=\beta 0+\beta 1=\text{environment}‐\text{oriented practices}. \end{array}$$

The linear regression result shows that there was a significant positive relationship between EOP and motivation at adj *r*^²^ = 0.180; the relationship was significant with *t*‐value = 8.386 at *P* ≤ 0.01.

Regression equation:

(8) $$\begin{array}{c} \left(\text{H}3\text{a}\right)\text{ Motivation}=\beta 0+\beta 1=\text{environment}‐\text{oriented practices}. \end{array}$$

## 6. Implication and Limitation

The generalizability of this study can extend to the other agriculture and primary production industries' workers in Pakistan and even beyond in other developing countries' rural agriculture workers. There are three major limitations of this study. First, the numbers of certified mango farms are limited in Pakistan. Second, this study was solely conducted in the Punjab province. Although the region is a major contributor to the national mango production, there was a significant mango cluster in the Sindh province which has been excluded from this study. The Sindh and Punjab provinces are different; their history, culture, and language are distinctive from each other. Third, the study does not include noncertified farms to establish a comparison. There were several mango farms in the region, which were not certified. It was possible to collect the data from those farms to make a comparative analysis between certified and noncertified farms. However, this study has ignored those farms and solely collects the data from the certified farms. It is not known if the GlobalGAP inspired the other smaller nonparticipating farmers or not.

In the Punjab province, the GlobalGAP standard has a successful case of uplifting the province's citrus industry [41], and the standard is also working with other growers such as sugarcane and cotton growers, but this study has limited its scope only to the mango industry. The findings of this research, however, can be generalised beyond the Punjab mango cluster to wider global rural agriculture workers in developing countries as they encounter similar challenges discussed and explored in this study.

The in-depth literature review revealed that there are many different definitions for CSR, and also the use of this term varied according to the themes of previous studies. This particular study purely focused on the role of private standards in ensuring compliance in developing countries. Private standards have specific codes and framework, but in this study, the focus was on CSR compliance. This study is based on the GlobalGAP (as a private) standard and its role in ensuring CSR compliance in the Pakistani mango industry. It is also evident from the existing literature that private standards are largely based on CSR-related issues and their prime role is to ensure compliance with CSR practices in the complex supply chains (Pilbeam et al., 2012; Sodano et al., 2008; [42]). Thus, regardless of GlobalGAP's emphasis on traceability and food safety, the standard does promise to ensure that minimum CSR requirements (social and environmental issues) have been met by the certified growers and producers.

## 7. Conclusion

Herzberg's two-factor theory had never been tested with rural agricultural workers. It is also critical to remember that there is a huge difference between the rural population of advanced countries and the rural population of developing countries. The participants of this study have acknowledged that GAP resulted in establishing a better understanding of social and environmental issues and responsibility. The workers also acknowledged that the working conditions had been improved at the certified farms.

The contributions of this research are fivefold. Firstly, this research has given an insight into the good agriculture practice initiatives of the Pakistani mango industry as it is the first ever study that has been conducted on this particular population. The approach was also different from other studies that have been carried out in other regions. Secondly, this research has contributed to knowledge as it shows a significant positive relationship between CSR practices for good agriculture and rural workers' motivation and job satisfaction. Thirdly, the research also identifies the particular practices that affect the workers' motivation the most. The workforce-oriented practices were found to be the most important factor followed by the environmental practices. Surprisingly, the society-oriented practices were found least significant to predict the rural workers' motivation. Fourthly, the findings of this research are equally important for practitioners and policymakers, as they are important for the academia. These findings have bridged a significant knowledge gap and also answer many questions that are part of a debate these days regarding the usefulness and benefits of private standards. Lastly, it puts forward recommendations for future research agenda that will help to spread the good agriculture practices across the entire agriculture industry in Pakistan and beyond.

The intention of this research was to examine the Pakistani mango industry and the role of GlobalGAP in ensuring CSR compliance at the certified farms. The rural workers have been ignored previously, and the phenomenon of rural workers' motivation is a new term that has been introduced in this research. There is a need to review the policy to address the issues of those workers. These workers are the strongest asset as 64% of the Pakistani population lives in rural areas, and 68% of them work in the agriculture sector; the sector also contributes 21.6% of the total country GDP [43]. The country, however, has much more potential, and it is critical for all the stakeholders to play their part in transforming the agriculture industry.

### 7.1. Future Research

There is a huge divide in what private standards such as the GlobalGAP are eyeing for the future, including the scientific ways and innovations to improve food security and sustainability. The agenda was revealed during the GlobalGAP Summit in Amsterdam on 27^th^ September 2016. The stakeholders on the other side are still confused and unclear on how to relate the need for private governance with their existing working atmosphere. While the academic and professional bodies are shifting their focus on future trends such as materiality index, collaborations, circular economy, and big data, the critical questions remained unanswered as to how CSR codes can change or rather transform the working conditions in developing countries. This study found that thousands of new jobs had been created at the certified mango farms and the pack houses, thus associated with poverty reduction. Pakistan is a hugely populated country, and that is why the difference GlobalGAP made is not clearly visible. However, there has been a significant reduction in poverty in the immediate surroundings of the certified farms.

Considering the findings from this study, along with the suggestions from the Brundtland Report, the Rio summit, and the sustainable development goals (SDGs), it was clear that the basic questions about poverty, human rights, inequality, and gender discrimination remained the top priorities for the world's leaders. The findings of this study look relevant and can be used as a base point to start exploring such complicated areas. With all these facts, future studies may try to investigate the following research questions:

1. Is it possible to tackle social injustice and inequality through private governance?
2. Can private standards play a role in building collaborations among the stakeholders in developing countries?

The element of life expectancy appeared as an unexpected finding of this research. It will be of great value if further research considers this area in order to examine the impacts of good agriculture practices on rural life expectancy in developing countries.

## 8. Recommendations

The recommendations have been made for the policies and the existing practices.

### 8.1. Stakeholder Engagement

Notwithstanding the success of the certified Pakistani mango growers in the western supermarkets, the numbers of certified mango farms are declining in the country. During the field trips, some of the growers expressed grievances and their intentions to cancel the subscription. The main cause of such grievances was the lack of communication and the absence of stakeholder engagement. After the implementation and issuance of the certificate, GlobalGAP failed to engage with the growers, and the growers were left relying on the local audit firms which were appointed by the GlobalGAP. There are many ways in which the GlobalGAP can actively interact with the participants, for example, through monthly or quarterly newsletters, through social media, or by launching a dedicated portal for the growers where they can post questions and queries.

### 8.2. Highlighting the Cause

The success of private standards has been vastly associated with the financial gains for the participating producers and growers, especially regarding gaining lucrative deals with the foreign supermarkets. Although the GlobalGAP was initiated by the supermarkets and retail groups in the EU, it was designed to address the global issues of ethical trading. The growers who were found to be less motivated during the field trips were those who failed to secure any sound deal with the EU supermarkets. There is a need for the GlobalGAP and other similar initiatives to promote the cause among the growers in developing countries.

### 8.3. Human Capital

The findings revealed that workforce-oriented practices were the most important factor for the workers. Thus, there is a need to focus on the workers. Currently, the GlobalGAP requires a minor must for the clauses related to workers. There is a need to make them a major must as with the environment and traceability factors. According to UNDATA (2014), 44% of the population in Pakistan was associated with agriculture, and in the whole South Asia, that was 45.4%; therefore, there is a need for serious reforms. The role of governments is critical as they can build on the existing work done by the GlobalGAP and other global players in the region. This study discussed the expertise of the mango workers and how they benefited from training and coaching at the certified farms. The study further found how that expertise helps the growers (the employers) in minimising the losses and enhancing the production volume. If such expertise can be transferred onto rural workers across the country, the agriculture sector shall do wonders.

### 8.4. Contribution to Knowledge

The literature on CSR and private standards in Pakistan is still inchoate. This research has filled this knowledge gap through empirical data. This study has made a methodological contribution, empirical contribution, theoretical contribution, and contribution to practice.

#### 8.4.1. Methodological Contribution

This study has produced a framework to observe the usefulness of private standards' codes or CSR initiatives in the context of developing countries. The framework has allowed the researcher to involve maximum participation from all involved stakeholders. From government representatives to NGO officials, and from growers and exporters to bottom-tier workers, every stakeholder has participated in this study. A combination of mixed methods (qualitative and quantitative) and multimethods (individual interview, focus groups, and observation) provided flexibility to explore almost all the segments of the GlobalGAP episode and the Pakistani mango industry transformation. The study further produced an instrument to test the rural workers' motivation against CSR practices by the help of combining CSR practices and motivation theory.

#### 8.4.2. Empirical Contribution

It was the first study of its kind that has ever been conducted in Pakistan. There were several types of research that have been carried out to explore the private agro standards in Africa and South America. Previous studies considered private standards as an extra burden and barrier to the growers in the poorer developing countries, but this study has revealed that there were options available for the growers to access funds that were supplied by the donors and exporters. The majority of the studies have been conducted to examine the financial benefits of private standards in terms of opening up new markets, access to supermarkets, and low transaction costs. This study, however, argued that private standards were not made solely to ensure financial gains for the participating producers and growers but were also designed to ensure food safety, environment protection, worker wellbeing, and sustainability, and therefore, they should be tested against these values too.

The empirical qualitative studies are rare in this particular category, and this study has attempted to enrich the knowledge about the relationship between CSR and performance in primary production industries. The rural workers in all developing countries face similar challenges including poverty, inequality, discrimination, poor health and education facilities, child labour, human rights violation, and hazard working conditions. The United Nations' sustainable development goals (SDGs) were also designed with a view to tackle these critical and alarming issues. This study has provided an insight into the implications of addressing these issues and has argued that initiatives such as GAP can pave the way to achieve those goals.

The researcher has adopted a unique approach in involving the participation of all the stakeholders in the Pakistani mango industry, and this approach has provided an insight into the complications and elusiveness in decision-making. The study has contributed to the ongoing debate about the verifiable or quantifiable benefits of private standards. This study was the first to test the relationship between private governed good agricultural practice codes and workers' motivation.

#### 8.4.3. Theoretical Contribution

The contribution to the theory of motivation has emerged through this research. The framework was based on Herzberg's two-factor theory of motivation to test the rural workers' motivation. Herzberg had suggested that the presence of hygiene factors can only avoid the element of dissatisfaction but cannot motivate the employees. The population he tested were urban, white collared employees such as accountants and bankers. However, when the same hygiene factors were tested on the poor rural workers, they were found to be strongly correlated with their motivation. This new theoretical finding of rural workers' motivation can be generalised to all rural workers elsewhere in the world, as they face similar challenges.

#### 8.4.4. Contribution to Practice

This study also contributes to the policy for approaching and pursuing social and environmental governance in developing countries. The synergistic model can be adopted and used for implementation policies in developing countries. The suggestions highlighted in this study should provide insight into policymaking that could help the growers in developing countries to better understand the importance of social and environmental issues and how compliance can affect their workforce, something which is a key asset for the growers. The current practice is largely focused on the financial returns for the growers, and when they fail to establish any engagement with the western supermarkets, they become demotivated easily. The financial gain is important and will occur in a more sustainable way if the growers commit to compliance with full motivation.

### 8.5. Moving Forward

The concept of compliance is an organisational practice that travels from top tier to bottom tier, in the ideal circumstances. The leadership and managers are expected to set the standards and also to coach the team. Compliance with international CSR codes of practice has opened up opportunities for growers in developing countries. There are several success stories and case studies to convince the stakeholders, especially in Africa and South America. It is a relatively new and uncommon phenomenon in Southeast Asia, particularly in Pakistan.

The study also suggested the following objectives for private standards to improve participation from the producers and growers in developing countries:

- Involve the growers and producers in policymaking
- Make this easier and convenient for a grower to start with basic requirements
- Motivate them instead of pressurising them
- Less emphasis on financial gain and more on productivity and sustainability
- Keep a regular interaction with the certified growers through newsletters, memos, or bulletins
- Share innovation, success stories, and latest researches with them
- Provide them access to the online resources
- Establish an efficient contact point to deal with complaints, queries, suggestions, or requests

## Figures and Tables

**Table 1:** Deductive and inductive approaches.

Deduction emphasis	Induction emphasis
Scientific principles	Gaining an understanding of the meanings humans attach to events
Moving from theory to data	A close understanding of the research context
The need to explain causal relationships between variables	The collection of qualitative data
The collection of quantitative data	A more flexible structure to permit changes of research emphasis as the research progresses
The application of controls to ensure validity of data	A realisation that the researcher is part of the research process
The operationalisation of concepts to ensure clarity of definition	Less concern with the need to generalise
A highly structured approach	
Researcher independence of what is being researched	
The necessity to select samples of sufficient size in order to generalise conclusions	

Source: [31].

**Table 2:** Kaiser-Meyer-Olkin adequacy test.

Kaiser-Meyer-Olkin measure of sampling adequacy	0.800
Bartlett's test of sphericity approx. chi square	6815.869
Df	325
Sig.	0.000

**Table 3:** Research paradigms.

Philosophical assumption	Positivism	Postpositivism	Critical theory	Constructivism
Ontology	Native realism: real reality exists but is apprehendable. It is conventionally summed up in time and context-free generalisations and is based on cause-effect laws.	Critical realism: real reality but only imperfectly and probabilistically apprehendable.	Historical realism: virtual reality shaped by social, political, cultural, economic, ethnic, and gender values, crystallised over time.	Relativism: local and specific constructed realities.
Epistemology	Dualist/objectivist; findings true.	Modified dualist/objectivist; critical tradition/community; findings probably true.	Transactional/subjective; value mediated findings.	Transactional/subjectivist; create findings.
Methodology	Experimental/manipulative; verification of hypotheses; chiefly quantitative methods.	Modifies experimental/manipulative; critical multiplism; falsification of hypotheses; may include qualitative methods.	Dialogic/dialectical.	Hermeneutical/dialectical.

Source: [32].

**Table 4:** Communalities.

	Initial	Extraction
WFOP1	0.822	0.573
WFOP2	0.831	0.758
WFOP3	0.641	0.588
WFOP4	0.572	0.315
WFOP5	0.759	0.634
WFOP6	0.666	0.600
WFOP7	0.514	0.480
WFOP8	0.765	0.491
SOP1	0.406	0.430
SOP2	0.594	0.377
SOP3	0.614	0.560
SOP4	0.710	0.703
EOP1	0.679	0.549
EOP2	0.772	0.636
EOP3	0.775	0.858
EOP4	0.632	0.431
JS1	0.502	0.362
JS2	0.900	0.878
JS3	0.810	0.696
JS4	0.783	0.720
M1	0.900	0.836
M2	0.830	0.703
M3	0.926	0.905
M4	0.845	0.701
M5	0.803	0.731
M6	0.766	0.708

**Table 5:** Likert scale.

Strongly agree	Agree	Neither agree nor disagree	Disagree	Strongly disagree
5	4	3	2	1

**Table 6:** Cronbach's alpha for all variables.

Cronbach's alpha	*N* of items
0.924	26

**Table 7:** Cronbach's alpha for independent variables (CSR practices).

Cronbach's alpha	No. of items
0.822	16

**Table 8:** Item-total statistics for all variables.

	Scale mean if item deleted	Scale variance if item deleted	Corrected item-total correlation	Cronbach's alpha if item deleted
WFOP1	111.62	132.985	0.577	0.922
WFOP2	112.09	124.683	0.737	0.918
WFOP3	112.50	127.737	0.484	0.924
WFOP4	111.68	136.409	0.388	0.924
WFOP5	112.12	123.023	0.759	0.918
WFOP6	111.85	133.414	0.570	0.922
WFOP7	111.72	138.156	0.245	0.925
WFOP8	112.37	121.389	0.684	0.920
SOP1	111.59	139.296	0.179	0.926
SOP2	111.60	141.440	-0.045	0.927
SOP3	111.68	137.476	0.326	0.924
SOP4	111.81	131.032	0.529	0.922
EOP1	111.52	138.987	0.270	0.925
EOP2	111.73	134.275	0.488	0.923
EOP3	111.62	137.385	0.370	0.924
EOP4	111.61	138.493	0.241	0.925
JS1	112.91	126.727	0.508	0.924
JS2	112.14	123.253	0.833	0.916
JS3	111.76	131.180	0.788	0.919
JS4	111.63	136.552	0.444	0.923
M1	112.11	123.200	0.806	0.917
M2	112.15	125.968	0.683	0.919
M3	112.29	116.671	0.889	0.915
M4	112.07	124.065	0.743	0.918
M5	112.05	127.816	0.624	0.920
M6	111.73	132.089	0.676	0.921

**Table 9:** Item-total statistics for independent variable (CSR practices).

	Scale mean if item deleted	Scale variance if item deleted	Corrected item-total correlation	Cronbach's alpha if item deleted
WFOP1	68.48	29.844	0.566	0.805
WFOP2	68.95	25.889	0.730	0.788
WFOP3	69.35	27.557	0.430	0.818
WFOP4	68.53	31.373	0.396	0.815
WFOP5	68.98	25.368	0.726	0.787
WFOP6	68.71	29.915	0.582	0.805
WFOP7	68.58	31.813	0.327	0.818
WFOP8	69.22	24.537	0.650	0.797
SOP1	68.45	32.826	0.182	0.824
SOP2	68.46	33.544	0.024	0.829
SOP3	68.53	32.066	0.301	0.819
SOP4	68.66	29.101	0.485	0.809
EOP1	68.37	32.470	0.337	0.819
EOP2	68.58	30.892	0.404	0.814
EOP3	68.47	32.225	0.303	0.819
EOP4	68.46	31.926	0.346	0.818

**Table 10:** Cronbach's alpha for job satisfaction.

*N* of items	Cronbach's alpha
4	0.677

**Table 11:** Cronbach's alpha for motivation.

Reliability statistics	
Cronbach's alpha	*N* of items
0.911	6

**Table 12:** Item-total statistics for job satisfaction.

	Scale mean if item deleted	Scale variance if item deleted	Corrected item-total correlation	Cronbach's alpha if item deleted
JS1	13.67	2.464	0.440	0.695
JS2	12.91	2.499	0.691	0.426
JS3	12.52	3.723	0.654	0.553
JS4	12.39	4.689	0.268	0.714

**Table 13:** Item-total statistics for motivation.

	Scale mean if item deleted	Scale variance if item deleted	Corrected item-total correlation	Cronbach's alpha if item deleted
M1	21.70	15.227	0.778	0.891
M2	21.73	15.389	0.772	0.892
M3	21.88	13.378	0.809	0.891
M4	21.66	14.633	0.850	0.880
M5	21.64	15.996	0.722	0.899
M6	21.32	18.369	0.670	0.912

**Table 14:** Combined descriptive data.

	Workforce-oriented CSR	Society-oriented CSR	Environment-oriented CSR	Job satisfaction	Motivation	
*N*						
Valid	316	316	316	316	316	
Missing	0	0	0	0	0	
*Mean*	35.2184	18.9146	19.1203	17.1614	25.9842
Std. error of mean	0.25416	0.06233	0.06853	0.13033	0.26342
Median	36.0000	19.0000	20.0000	18.0000	28.0000
*Mode*	40.00	20.00	20.00	20.00	30.00
*Std. deviation*	4.51802	1.10797	1.21816	2.31679	4.68260
Variance	20.412	1.228	1.484	5.368	21.927	
Range	18.00	4.00	4.00	8.00	17.00	
Minimum	22.00	16.00	16.00	12.00	13.00	
*Maximum*	40.00	20.00	20.00	20.00	30.00	
Sum	11129.00	5977.00	6042.00	5423.00	8211.00	

## Data Availability

The data is not available in the main manuscript, and result analysis is provided through tables and figures. All data are associated with its findings.

## References

[1] UNIDO (2015). Global value chains and development UNIDO‘s support towards inclusive and sustainable industrial development. Vienna International Centre, Austria. United Nations, (1987). *Our Common Future-Brundtland Report*.

[2] Amekawa Y. (2009). Reflections on the growing influence of good agricultural practices in the global south. *Journal of Agricultural and Environmental Ethics*.

[3] Jones P., Comfort D. (2003). Retailing fair trade food products in the UK. *British Food Journal*.

[4] UNEP (2011). *Corporate Social Responsibility and Regional Trade and Investment Agreements*.

[5] Lund-Thomsen P., Nadvi K. (2010). Global value chains, local collective action and corporate social responsibility: a review of empirical evidence. *Business Strategy and the Environment*.

[6] Kuwornu J. K. M., Mustapha S. (2013). Global GAP standard compliance and smallholder pineapple farmers‘access to export markets: implications for incomes. *Journal of Economics and Behavioural Studies*.

[7] International Trade Centre (2011). *The Impacts of Private Standards on Producers in Developing Countries*.

[8] Lund-Thomsen P., Nadvi K. (2010). Clusters, chains and compliance: corporate social responsibility and governance in football manufacturing in South Asia. *Journal of Business Ethics*.

[9] Robinson P. K. (2010). Responsible retailing: the practice of CSR in banana plantations in Costa Rica. *Journal of Business Ethics*.

[10] Schuster M., Maertens M. (2013). Do private standards create exclusive supply chains? New evidence from the Peruvian asparagus export sector. *Food Policy*.

[11] Elkington J. (1998). *Cannibals with forks: the triple bottom line of 21st century business*.

[12] UNIDO (2014). What is CSR. http://www.unido.org/csr/o72054.html.

[13] European Commission (2011). Communication from the Commission to the European Parliament, the Council, the European Economic and Social Committee and the Committee of the Regions. *A Renewed EU Strategy 2011-14 for Corporate Social Responsibility*.

[14] Boehe D. M., Barin Cruz L. (2010). Corporate social responsibility, product differentiation strategy and export performance. *Journal of Business Ethics*.

[15] Carroll A. B. (1979). A three-dimensional conceptual model of corporate performance. *Academy of Management Review*.

[16] Habisch A., Jonker J., Wegner M., Schmidpeter R. (2005). *Corporate Social Responsibility across Europe*.

[17] European Commission (2007). Enterprise and industry directorate-general. *CSR and Competitiveness European SMEs‘Good Practice: Consolidated European Report*.

[18] Friedman M. (1962). *Capitalism and Freedom*.

[19] World Bank Working Paper (2005). *Changing European Public and Private Food Safety and Quality Requirements: Challenges for Developing Country Fresh Produce and Fish Exporters: European Union Buyers Survey*.

[20] Financial Times (2007). https://www.ft.com/content/abfb%200a28-09fd-11dc-93ae-000b5df10621/.

[21] Jones P., Comfort D., Hillier D., Eastwood I. (2005). Corporate social responsibility: a case study of the UK‘s leading food retailers. *British Food Journal*.

[22] Loconto A. (2014). Sustaining an enterprise, enacting sustainabilitea. *Science, Technology, & Human Values*.

[23] Thompson L. J., Lockie S. (2013). Private standards, grower networks, and power in a food supply system. *Agriculture and Human Values*.

[24] Robbins S. P., Coulter M. (2012). *Management*.

[25] Turyakira P., Venter E., Smith E. (2014). The impact of corporate social responsibility factors on the competitiveness of small and medium-sized enterprises. *South African Journal of Economic and Management Sciences*.

[26] De Neve G. (2014). Fordism, flexible specialization and CSR: how Indian garment workers critique neoliberal labour regimes. *Ethnography*.

[27] Van Knippenberg D. (2000). Work motivation and performance: a social identity perspective. *Applied Psychology*.

[28] Kim C. H., Scullion H. (2013). The effect of corporate social responsibility (CSR). On employee motivation: a cross-national study. *Poznań University of Economics Review*.

[29] FAO Report (2014). *Developing Sustainable Food Value Chains Guiding Principles*.

[30] Scotland J. (2012). Exploring the philosophical underpinnings of research: relating ontology and epistemology to the methodology and methods of the scientific, interpretive, and critical research paradigms. *English Language Teaching*.

[31] Saunders M., Lewis P., Thornhill A. (2009). *Research Methods for Business Students (5th Edition)*.

[32] Guba E. G., Lincoln Y. S., Denzin N. K., Lincoln Y. S. (1994). Competing paradigms in qualitative research. *Handbook of Qualitative Research*.

[33] Burrell G., Morgan G. (1979). *Sociological Paradigms and Organizational Analysis*.

[34] Lincoln Y. S., Guba E. G. (1985). *Naturalistic Inquiry*.

[35] Eriksson P., Kovalainen A. (2008). *Qualitative Methods in Business Research*.

[36] Gray D. E. (2013). *Doing Research in the Real World (3rd Edition)*.

[37] Bryman A., Bell E. (2015). *Business Research Methods (4th Edition)*.

[38] Sandelowski M. (2000). Focus on research methods combining qualitative and quantitative sampling, data collection, and analysis techniques in mixed-method studies. *Research in Nursing & Health*.

[39] Campbell D. T., Stanley J. C. (1963). Experimental and quasi-experimental designs for research. *Handbook of Research on Teaching*.

[40] Hansen H., Trifkovic N. (2014). Food standards are good - for middle-class farmers. *World Development*.

[41] FAO (2013). *Agribusiness Public-Private Partnerships – A Country Report of Pakistan*.

[42] Tallontire A. (2007). CSR and regulation: towards a framework for understanding private standards initiatives in the agri-food chain. *Third World Quarterly*.

[43] World Bank (2014). Project appraisal document on a proposed credit: to the Islamic Republic of Pakistan for a Sindh Agricultural Growth Project. *Sustainable Development Department Agriculture*.

